# Impact of pharmacological sGC stimulation with Riociguat in experimental models of liver disease regression

**DOI:** 10.1007/s12072-026-11086-4

**Published:** 2026-04-15

**Authors:** Katharina Bonitz, Philipp Königshofer, Henriette Horstmeier, Thomas Sorz-Nechay, Vlad Taru, Katharina Bareiner, Oleksander Petrenko, Benedikt Simbrunner, Benedikt Silvester Hofer, Georg Kramer, Katja Sommer, Kerstin Zinober, Katharina Regnat, Hubert Scharnagl, Peng Sun, Eric Simon, Stefan Kauscke, Michael Trauner, Thomas Reiberger, Philipp Schwabl

**Affiliations:** 1 Division of Gastroenterology and Hepatology, Department of Medicine III, Medical University of Vienna, Waehringer Guertel 18-20, 1090 Vienna, Austria; 2 Hepatic Experimental Hemodynamic Lab (HEPEX) Lab, Medical University of Vienna, Vienna, Austria; 3 Christian-Doppler Laboratory for Portal Hypertension and Liver Fibrosis, Medical University of Vienna, Vienna, Austria; 4 CeMM Research Center for Molecular Medicine of the Austrian Academy of Sciences, Vienna, Austria; 5 Clinical Institute of Medical and Chemical Laboratory Diagnostics, Graz, Austria; 6 CardioRenalMetabolic Diseases Research, Boehringer Ingelheim Pharma GmbH & Co.KG, Biberach an Der Riss, Germany; 7 Computational Innovation, Boehringer Ingelheim Pharma GmbH & Co.KG, Biberach an Der Riss, Germany

**Keywords:** Liver fibrosis regression, Soluble guanylyl cyclase, Riociguat, Experimental fibrosis, Portal hypertension, Liver transcriptomics

## Abstract

**Background:**

Liver fibrosis and portal hypertension (PH) determine prognosis in chronic liver disease. In experimental models, stimulation of NO–sGC–cGMP signaling improved fibrosis, PH and inflammation. Since fibrosis and PH improve slowly after injury cessation, we investigated whether stimulating sGC activity accelerates their regression.

**Methods:**

Liver fibrosis was induced in C57BL/6 J mice by either carbon tetrachloride (CCl_4_; 2 µl/g, gavage 3x/week) for 12 weeks or thioacetamide (TAA; 150 mg/kg, intraperitoneal injections 3x/week) for 12 weeks, followed by 1 (R1) or 2 (R2) weeks of regression. Animals received the sGC stimulator Riociguat (RIO; 3 mg/kg, gavage 2x/day) during regression. Disease severity was assessed by portal pressure (PP), collagen proportionate area (CPA) and whole liver transcriptomics.

**Results:**

PH and fibrosis area peaked in the TAA model at 7.71 ± 0.57 mmHg PP and 3.87 ± 0.19% CPA; and in the CCl_4_ model at 9.06 ± 0.89 mmHg PP and 9.43 ± 2.59% CPA, respectively; followed by spontaneous regression at R2 (TAA: PP: 6.04 ± 0.52 mmHg, CPA: 2.90 ± 0.30%; CCl_4_: PP: 5.88 ± 0.52 mmHg, CPA: 6.66 ± 1.45%). RIO significantly increased hepatic cGMP levels (CCl_4_–R2: 5.89 ± 0.58 nmol/L, R2 + RIO: 12.41 ± 1.98 nmol/L). While fibrosis and PH regression were not significantly different, RIO treatment attenuated the hepatic pro-inflammatory gene signatures in both models and improved metabolic pathways on a transcriptional level.

**Conclusions:**

Riociguat increases hepatic cGMP bioavailability and mitigates inflammatory and metabolic gene signatures in two experimental models of regressive liver disease. While regression of liver fibrosis and PH was not accelerated by Riociguat, our results suggest beneficial effects of sGC stimulation during regressive liver disease.

**Graphical abstract:**

**Figure:**
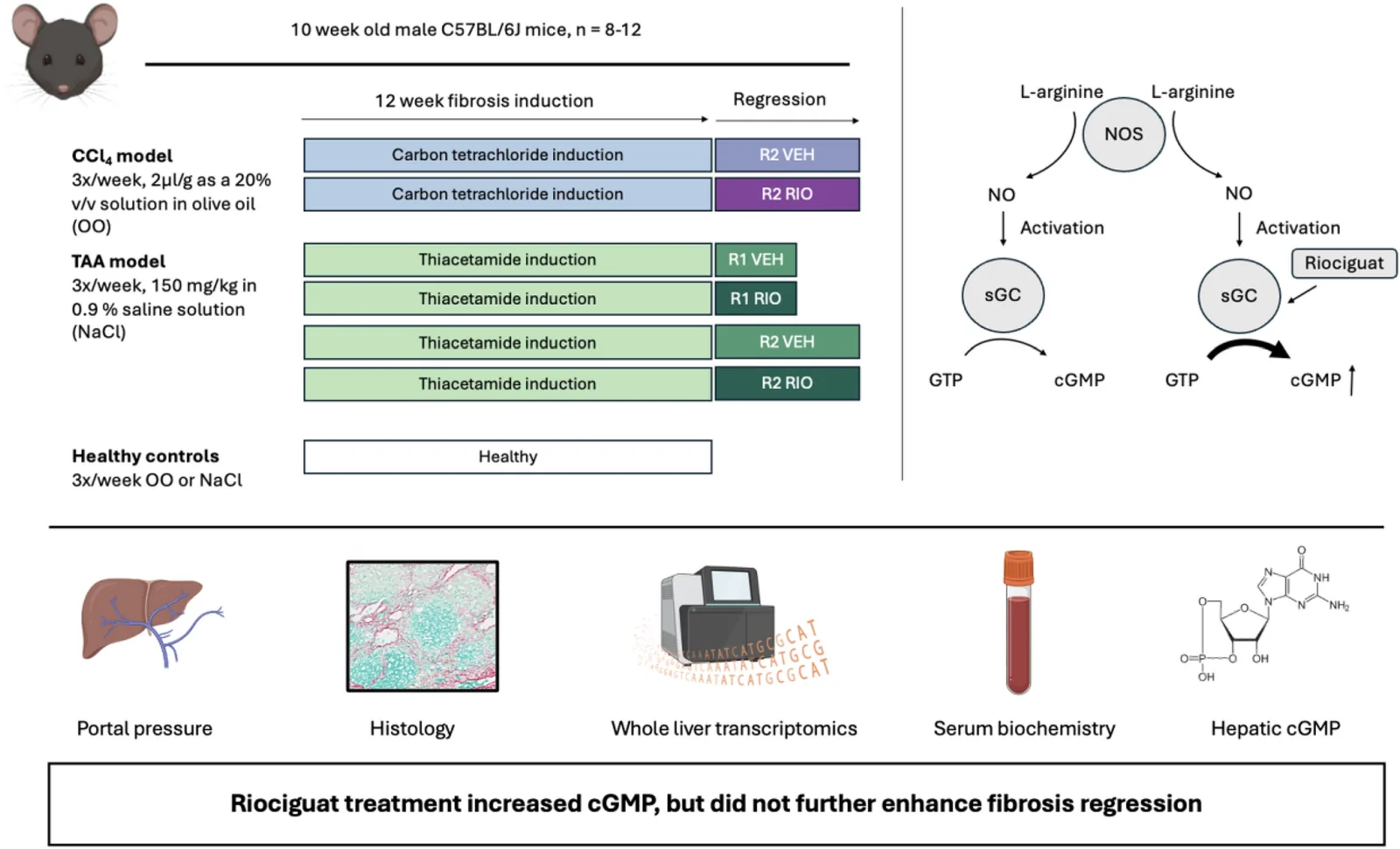

**Supplementary Information:**

The online version contains supplementary material available at 10.1007/s12072-026-11086-4.

## Introduction

Liver cirrhosis is the result of repetitive hepatic injury, which triggers inflammation and fibrogenesis, leading to scar formation and impaired hepatic function [1] A key pathophysiological mechanism is the transdifferentiation of hepatic stellate cells (HSCs) to myofibroblasts, that secrete extracellular matrix (ECM) components, which accumulate as fibrotic scar [2]. Moreover, liver sinusoidal endothelial cells (LSECs) undergo capillarization, marked by the disappearance of fenestration and development of a basement membrane [3]. These alterations increase vascular restriction and impair the fluid exchange between sinusoids and hepatocytes, further promoting fibrogenesis [4].

After cessation of hepatic injury, the reversal of fibrosis has been observed both in humans and in animal models [5, 6]. Fibrosis regression is characterized by a reduction in collagen proportionate area, coinciding with a decrease in PH and liver injury markers [7–9]. The observed changes in the liver microenvironment initially affect inflammatory pathways, allowing a switch from pro-fibrotic to pro-restorative macrophages, that degrade the ECM [10, 11]. In parallel, myofibroblasts become either quiescent or apoptotic and thus lose their fibrogenic activity [2, 12].

A promising axis for therapeutic intervention in chronic liver diseases appears to be the NO–sGC–cGMP pathway [13]. Canonically, nitric oxide (NO) is derived from endothelial (eNOS) and inducible nitric oxide synthases (iNOS). It is released from endothelial cells and activates the soluble guanylyl cyclase (sGC), which in turn catalyzes the reaction from GTP to cGMP [14]. Increased cGMP levels mediate the vasodilatory effect of NO, but also act anti-fibrotic and anti-inflammatory [15, 16]. Recognition of the therapeutic potential of this axis led to the development of numerous pharmaceutical approaches for the modulation of cGMP levels, including phosphodiesterase (PDE) inhibitors, sGC activators or sGC stimulators [17–20]. The latter are enhancing the sensitivity of sGC toward NO and, moreover, can also stimulate sGC in the absence of NO [14].

The sGC stimulator Riociguat (RIO) was first approved in a clinical setting for pulmonary hypertension in 2013 [21]. Subsequently, this drug class has gained increasing attention in the field of liver fibrosis and PH. First experimental studies using sGC stimulators and activators show promising outcomes, including a significant reduction in PP and liver fibrosis [18–20, 22]. These beneficial changes are accompanied by a marked reduction in liver inflammation, including NFkB signaling [20, 23]. In addition, increased sGC activity has been shown to induce LSEC differentiation and a reduction in capillarization, which in turn has led to a regressive-like phenotype in animal models [24].

So far, all experimental studies and first human trials [25] on enhancing sGC signaling have only been conducted in progressive liver disease and the question remains, whether increasing cGMP levels could accelerate liver repair in a setting of regressive liver disease.

## Materials and methods

### Animal models

All animal models were approved by the Austrian Government (BMBWF-V/3b/2020–0.398.007). The experiments were conducted with 10-week-old male C57BL/6 J mice. Animals were housed at the Center of Biomedical Research of the Medical University Vienna under controlled and standardized conditions (day/night cycle 12:12 h; room temperature 22 ± 2 °C, humidity 55 ± 10%) in conventional Makrolon type II-L cages in groups of 6–8 (littermates). The animal husbandry and hygienic standards were regularly monitored by local standard operating procedures according to the standards of care and maintenance of laboratory animals.

Liver fibrosis was induced by either oral gavage of CCl_4_ (3x/week, 2 µl/g as a 20% v/v solution in OO), or intraperitoneal injection of TAA (3x/week, 150 mg/kg in 0.9% saline solution (NaCl)) for a time period of 12 weeks, as previously described [26]. It was followed by either a 1 or a 2-week regression period, in which animals received either RIO treatment (2x/day, 3 mg/kg, in 0.5% Natrosol + 0.015% Tween-80 solution) or vehicle (2x/day, 0.5% Natrosol + 0.015% Tween-80 solution). The TAA model was conducted with a group size of n = 8 and the CCl_4_ model was studied with a group size of *n* = 12.

### Hemodynamic readouts

At the respective study endpoints, hemodynamics were evaluated under body weight-adjusted ketamine (100 mg/kg)/xylazine (7.5 mg/kg) anesthesia. PP was measured by direct cannulation of the portal vein using a 26G needle, connected via fine-pore polyethylene tubing (0.4 mm ID, REF: 800/100/140; Smiths Medical, Minneapolis, USA) to a pressure transducer (MLT0380/D ADInstruments, Colorado Springs, USA). Pressure was recorded using the ML870 Power Lab (ADInstruments). Subsequently, blood was withdrawn from the superior vena cava. Organ weights were documented and liver samples were harvested using a standardized lobe sampling for different molecular readouts.

### Histology

To evaluate liver fibrosis, the lateral left lobe was sliced into 1 mm sections, which were fixed in formaldehyde and embedded in paraffin wax. Tissue sections were stained with Picro-Sirius Red (PSR; Morphisto, Nr. 13425) and counterstained with 0.1% Fast-Green (FG; REF: 104,022, C.I.42053, Sigma-Aldrich, St. Louis, USA) to assess fibrosis. The stained slides were digitalized using a slide scanner at 40 × magnification (Slideview VS200; OLYMPUS, Shinjuku, Japan) and CPA was quantified (HALO® image analysis software, Indica Labs).

### Hepatic cGMP measurements

Hepatic cGMP levels were quantified by ultra-performance liquid chromatography coupled to tandem mass spectrometry (UPLC–MS/MS) as previously described [27]. Liver tissue samples were homogenized in 2% formic acid containing 2 mM IBMX at a ratio of 1:4 (w/v) and centrifuged at 13.000 rpm for 5 min at 4 °C. The resulting supernatant was combined with an internal standard (1 µM cGMP-^13^C,^15^N₂; #TRC-G837962, LGC Standards, Middlesex, United Kingdom) at a dilution ratio of 40:1.

A standard calibration curve was generated using defined concentrations of cGMP (#G7504, Merck, Darmstadt, Germany) spanning 0.2–1000 nM. Sample purification was carried out by solid-phase extraction using an Oasis WAX 96-well plate (#186002502, Waters Corporation, Massachusetts, USA). Analytes were eluted with 1% ammonium hydroxide in 95% methanol, followed by evaporation under a nitrogen stream at 45 °C. Dried samples were subsequently reconstituted in 0.1% formic acid prior to analysis.

Chromatographic separation was performed on an Acquity Premier HSS T3 column (#186009468, Waters Corporation, Massachusetts, USA) maintained at 40 °C. The mobile phase consisted of (A) water with 0.1% formic acid and (B) acetonitrile with 0.1% formic acid, applied in a gradient mode. Mass spectrometric detection was conducted on an AB Sciex 3Q MS 6500 + system (Sciex, Massachusetts, USA). Quantification of hepatic cGMP was achieved using multiple reaction monitoring (MRM), monitoring the ion transition m/z 346.2 → 152.1 for cGMP and m/z 349.1 → 155.1 for the isotopically labeled internal standard.

### Liver transcriptomics

Liver tissue was harvested from the median lobe, pre-frozen in methylbutane and snap-frozen in liquid nitrogen. For both models, liver tissue of *n* = 8 animals per group was sequenced. RNA extraction was performed using the RNeasy Mini Kit (Quiagen, Germany). Samples were submitted to the Biomedical Sequencing Facility of the CeMM Research Center for Molecular Medicine of the Austrian Academy of Sciences for further processing. Total RNA was used to generate stranded, poly-A selected libraries, which were sequenced on the Illumina NovaSeq platform using 2 × 50 bp paired-end reads. Reads were aligned to Mus musculus GRCm38/mm10 Reference Genome Assembly. Sequencing depth varied significantly between studies with approximately 15 million reads per sample for the TAA model and 40 million reads per sample for the CCl_4_ model. After alignment and filtering this resulted in 7537 genes for the TAA and 17360 genes in the CCl_4_ model. Genes without variance between samples and detected in less than 10% of the samples were excluded from downstream analysis. Downstream analysis of the acquired data was performed separately on both models.

Differential expression analysis (DEA) was conducted using the DESeq2 package [28] after variance stabilizing transformation for each model independently. For pairwise comparisons, the Wald test was applied with the factorial design ∼ condition. Principal component analysis (PCA) was performed using the PCAtools package. Gene set enrichment analysis was performed using the clusterProfiler package and MsigDB with Hallmark pathways as references. To understand which genes were affected by RIO treatment, pseudo-temporal analysis was performed using the ImpulseDE package [29]. The top 50 genes were evaluated based on *q* value. Genes were then selected based on their trajectories, with the aim to identify genes, which were beneficially regulated by treatment. Statistics were assessed based on padj with a cutoff of 0.05 unless otherwise stated in the figure legend.

### Statistical analysis

Phenotypic data from this study are expressed as mean ± standard deviation and statistical assessment was performed using GraphPad Prism (GraphPad Software, San Diego, CA) and R 4.3.1 (R Core Team, R Foundation for Statistical Computing, Vienna, Austria). Depending on the presence of a normal distribution as assessed by the Shapiro–Wilk test and a visual inspection of density plots, either a one-way ANOVA with Tukey’s post hoc analysis was performed if all groups were normally distributed, a Welch ANOVA with Dunnett’s T3 post hoc analysis was performed if one group did not pass the normality test, and a Kruskal–Wallis test and Dunn’s post hoc analysis were used for data where more than one group was not normally distributed. The significance level was set at 0.05 (95% confidence interval).

### Generative AI and AI-assisted technologies in the writing process

During the preparation of this work the authors used ChatGPT to improve text quality and structure. After using this tool, the authors reviewed and edited the content as needed and took full responsibility for the content of the publication.

## Results

### Spontaneous fibrosis regression for 2 weeks closes therapeutic windows

**Fig. 1:**
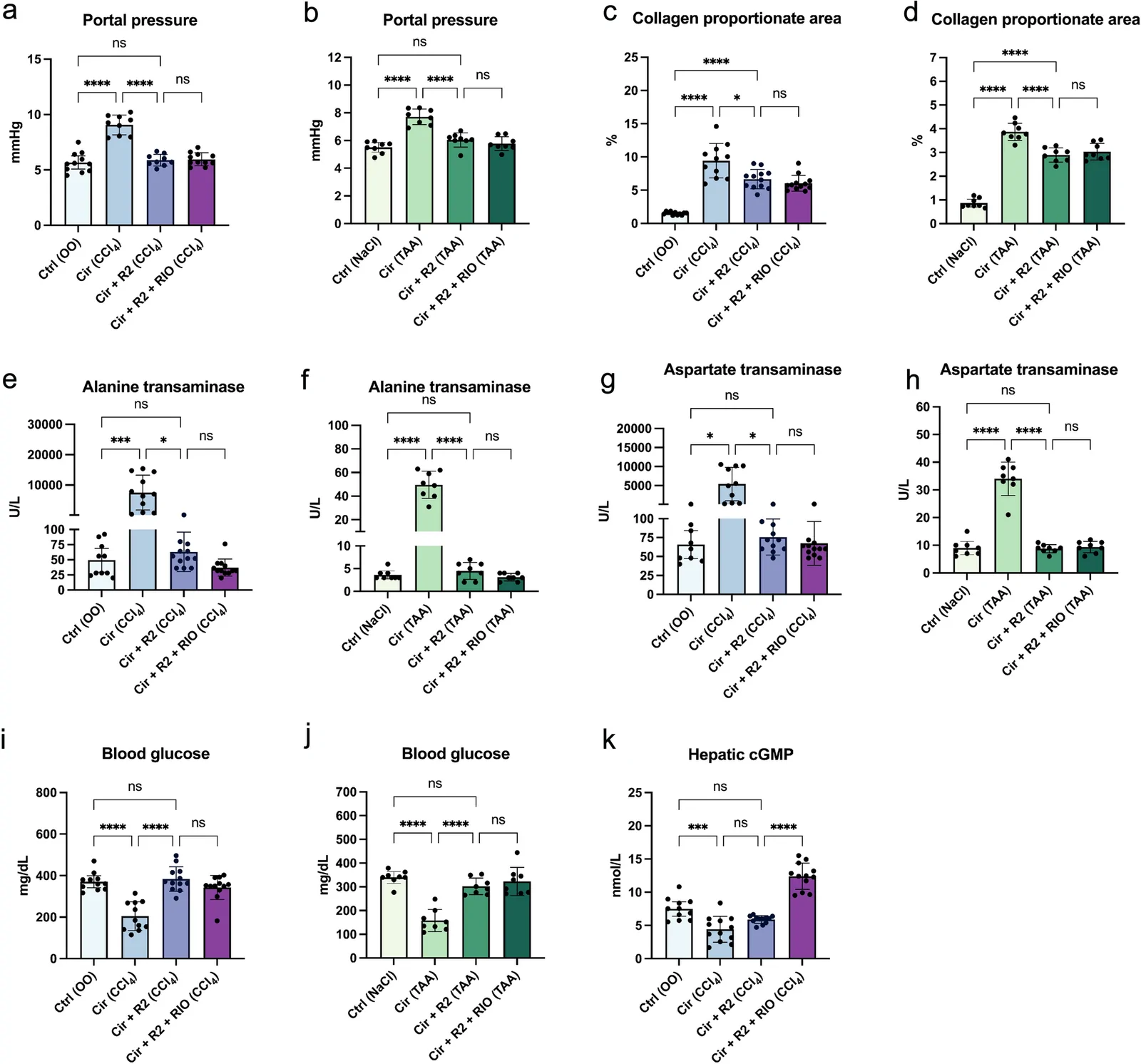
Main in vivo readouts in two liver disease regression models, including a Riociguat treatment subgroup. **A** Portal pressure (CCl_4_ model). **B** Portal pressure (TAA model). **C** Collagen proportionate area (CCl_4_ model). **D** Collagen proportionate area (TAA model). **E** Serum alanine transaminases (CCl_4_ model). **F** Serum alanine transaminases (TAA model). **G** Serum aspartate transaminases (CCl_4_ model). **H** Serum aspartate transaminases (TAA model). **I** Blood glucose level (CCl_4_ model). **J** Blood glucose level (TAA model). **K** Hepatic cGMP level (CCl_4_ model). Statistics: one-way ANOVA with Tukey’s multiple comparison test, Welch ANOVA with Dunnett’s T3 multiple comparison test or Kruskal–Wallis test with Dunn’s multiple comparison test were used, after determination of normality by the Shapiro–Wilk test. Significance: *p < 0.05; **p < 0.01; ***p < 0.001; ****p < 0.0001. *OO* olive oil, *CCl*_*4*_ carbon tetrachloride, *TAA* thioacetamide, *R2* 2-week regression, *RIO* Riociguat

Disease induction led to the development of PH (Fig. 1A, B) and fibrosis, assessed by CPA (Fig. 1C, D) in both animal models. Further peak fibrosis presented with elevated liver injury markers alanine transaminase (ALT) (Fig. 1E, F) and aspartate transaminase (AST) (Fig. 1G, H) and reduced blood glucose (Fig. 1I, J). Moreover, increased liver-to-body weight ratio (Suppl. Figure 1 A, B) and spleen-to-body weight ratio (Suppl. Figure 1 C, D), as well as elevated serum bile acid levels (Suppl. Figure 1E, F) could be observed.

Spontaneous fibrosis regression for 2 weeks (R2) led to a reduction to baseline values for most inflammatory-driven readouts in both animal models. Therefore, the therapeutic window for RIO treatment was closed for PH, ALT, AST, blood glucose and serum bile acids. After 2 weeks of regression, fibrosis (CPA) remained significantly elevated compared to healthy controls in both models. However, RIO treatment did not further reduce fibrosis in these animals (Fig. 1C, D).

To evaluate intrahepatic sGC activity, cGMP levels were measured in the CCl_4_ model. In peak disease, a decline in hepatic cGMP could be observed (Fig. 1K). After 2 weeks of RIO treatment, a significant upregulation of hepatic cGMP levels could be measured.

### Riociguat treatment during 1 week of fibrosis regression

**Fig. 2:**
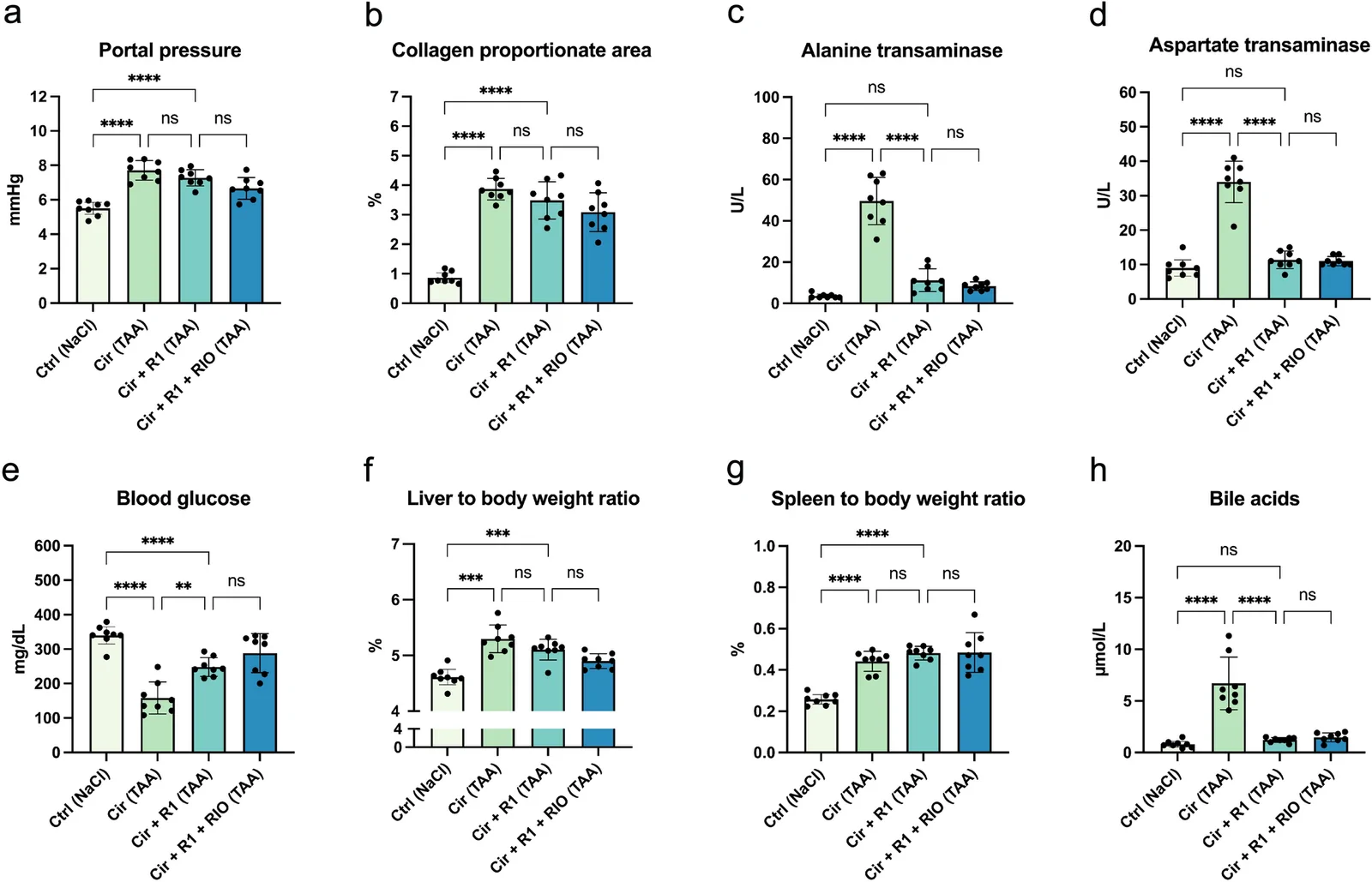
Main in vivo readouts in a short-term liver disease regression model. **A** Portal pressure. **B** Collagen proportionate area. **C** Serum alanine transaminases. **D** Serum aspartate transaminases. **E** Blood glucose level. **F** Liver to body weight ratio. **G** Spleen to body weight ratio. **H** Serum bile acids. Statistics: one-way ANOVA with Tukey’s multiple comparison test, Welch ANOVA with Dunnett’s T3 multiple comparison test or Kruskal–Wallis test with Dunn’s multiple comparison test were used, after determination of normality by the Shapiro–Wilk test. Significance: *p < 0.05; **p < 0.01; ***p < 0.001; ****p < 0.0001. *OO* olive oil, *CCl*_*4*_ carbon tetrachloride, *TAA* thioacetamide, *R1* 1-week regression, *RIO* riociguat

Two weeks of spontaneous regression in murine models of toxically induced liver disease led to fast resolution of PH and liver injury, thus closing the therapeutic window for RIO in regressive disease. Therefore, we further evaluated a shorter regression period of 1 week in the TAA model. After 1 week of natural regression, both PH and fibrosis were not significantly reduced compared to peak disease (Fig. 2A, B), while serum ALT and AST, as well as bile acids returned to baseline values (Fig. 2C, D, H). Blood glucose improved, but did not return to baseline within 1 week of regression (Fig. 2E). On the contrary, liver and spleen to body weight ratios did not change within 1 week of regression (Fig. 2F, G). RIO treatment during this 1 week of regression did not lead to significant changes in portal pressure, liver fibrosis or injury markers. Although numerical differences were observed in portal pressure, CPA, blood glucose, and liver to body weight ratio, these changes did not reach statistical significance (Fig. 2A, B, E, F).

### Changes of the hepatic transcriptome during fibrosis regression

To decipher the mechanisms underlying spontaneous regression and to evaluate if RIO treatment changes the hepatic transcriptome, even though no significant benefits were observed on the phenotypic level, we performed whole liver transcriptomics.

**Fig. 3:**
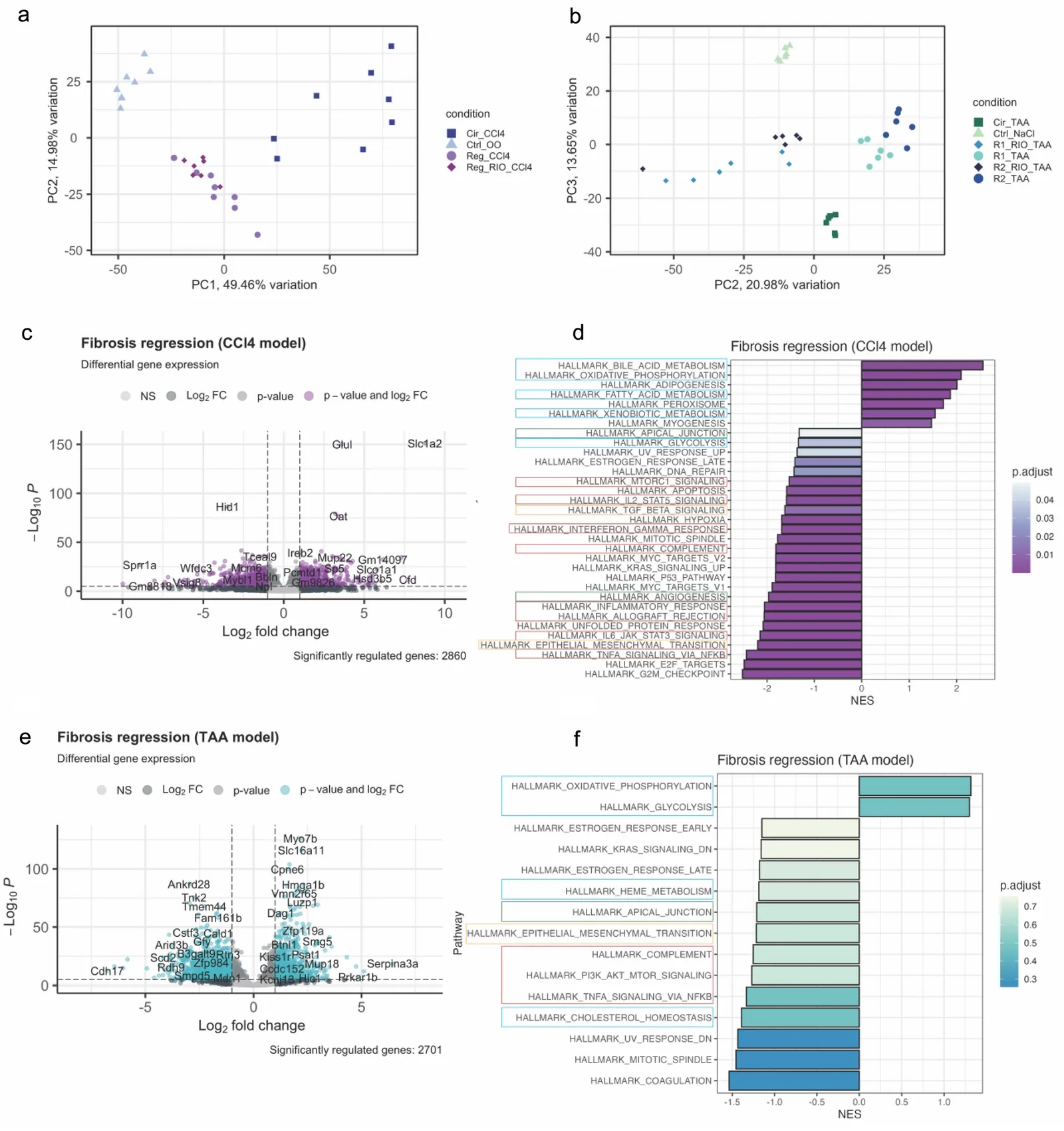
Hepatic transcriptomic profile during disease regression. **A** Principal component analysis (CCl_4_). **B** Principal component analysis (TAA). **C** Volcano plot of differentially regulated genes between fibrosis regression and peak fibrosis (CCl_4_ model). **D** Gene set enrichment analysis (GSEA) of differentially regulated genes in fibrosis regression, p.adjust cutoff: 0.05 (CCl_4_ model). **E** Volcano plot of differentially regulated genes between fibrosis regression and peak fibrosis (TAA model). **F** Gene set enrichment analysis (GSEA) of differentially regulated genes in fibrosis regression, p.adjust cutoff: 0.75 (TAA model). Color code in Figures **D** and **F**: blue—metabolism, red—inflammation, green—vascular remodeling/response, yellow—fibrogenesis. *CCl*_*4*_ carbon tetrachloride, *TAA* thioacetamide, *R1* 1-week regression, *R2* 2-week regression, *RIO* riociguat

PCA revealed distinct clustering of Healthy, Peak disease and regression in both models (Fig. 3A, B). In the CCl_4_ model, RIO-treated animals clustered together with spontaneous regression, whereas RIO-treated animals in the TAA model did not overlap with the spontaneously regressing counterparts.

In progressive liver disease we identified 1852 genes in the TAA and 5350 genes in the CCl_4_ model, which were differently regulated compared to healthy controls. Top hits included genes, which have previously been reported in liver fibrosis, such as *Mmp12*, *Col1a2* and *Timp2*. Gene set enrichment analysis revealed an upregulation of Epithelial Mesenchymal Transition (EMT) and inflammatory pathways, while metabolic pathways were downregulated in both models (Suppl. Figure 2A–D).

For the analysis of fibrosis regression in the TAA model the regression timepoints R1 and R2 were pooled. During regression, in the CCl_4_ model 2860 relevant genes could be identified, while in the TAA model 2701 genes significantly changed. Top genes included an upregulation of the ammonia- and lipid metabolism-controlling *Glul*, *Slc1a2*, and *Slc16a11* (Fig. 3C, F). In line, GSEA revealed an upregulation of metabolic function during regression in both models (Fig. 3D, F). Furthermore, EMT and inflammatory pathways were downregulated in the regression phase.

### Riociguat changes the hepatic transcriptome during fibrosis regression

**Fig. 4:**
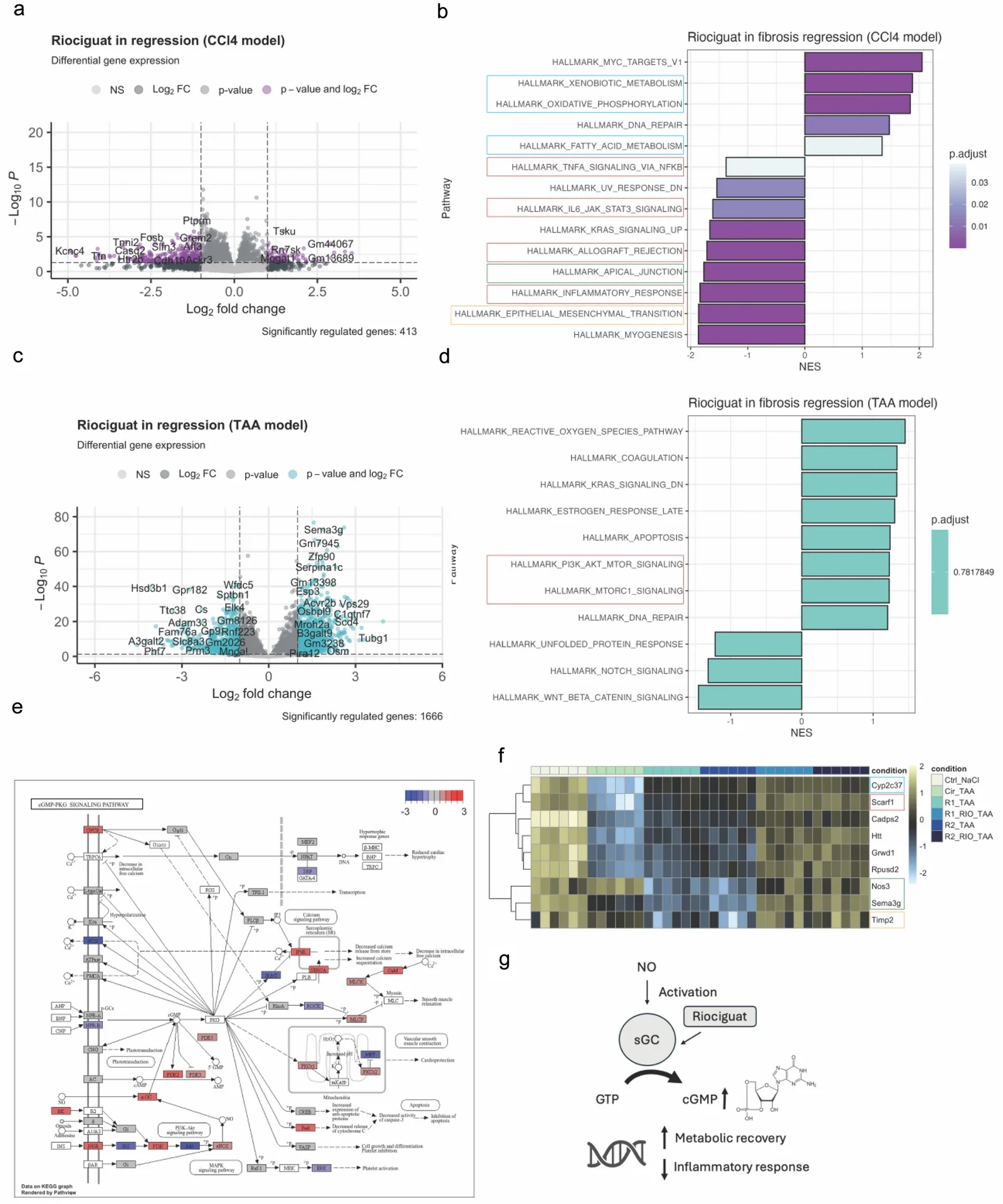
Effects of sGC simulation with Riociguat on the liver transcriptome during liver disease regression. **A** Volcano plot of differentially regulated genes between and RIO in regression and spontaneous regression (CCl_4_ model). **B** Gene set enrichment analysis (GSEA) of differentially regulated genes in RIO treatment, p.adjust cutoff: 0.05 (CCl_4_ model). **C** Volcano plot of differentially regulated genes between RIO in regression and spontaneous regression (TAA model). **D** Gene set enrichment analysis (GSEA) of differentially regulated genes in RIO treatment, p.adjust cutoff: 0.8 (TAA model). **E** Pathview analysis of differentially expressed genes in RIO treatment in regression compared to spontaneous regression for the cGMP–PKG pathway (TAA model). **F** Heatmap showing the top regulated genes (TAA model). **G** Graphical representation of RIO treatment in fibrosis regression. Color code in Figures **B**, **D** and **F**: blue—metabolism, red—inflammation, green—vascular remodeling/response, yellow—fibrogenesis. *CCl*_*4*_ carbon tetrachloride, *TAA* thioacetamide, *R1* 1-week regression, *R2* 2-week regression, *RIO* riociguat

RIO treatment affected expression of 413 genes in the CCl_4_ model and 1666 genes in the TAA model compared to spontaneous regression (Fig. 4A, C). In both models, improvements in metabolic pathways upon RIO treatment could be detected using GSEA (Fig. 4B, D). In the CCl_4_ model, the effect was more pronounced and statistically significant regarding regulation of xenobiotic metabolism, oxidative phosphorylation and fatty acid metabolism. Furthermore, in the CCl_4_ model RIO caused a downregulation of inflammatory pathways, such as the IL6–STAT3 axis and TNF signaling via NFkB. In addition, a downregulation of EMT was induced by RIO. Genes associated with vascular remodeling improved upon RIO treatment in regression (Suppl. Fig. 2 F).

Notably, the NO–sGC–cGMP pathway was differently affected by RIO in the two animal models. In the TAA group RIO treatment further stimulated the NO–sGC–cGMP pathway in regression (Fig. 4E), while it was mostly downregulated in the CCl_4_ group (Suppl. Fig. 2E). This observation aligns with a more pronounced effect of RIO treatment in the TAA group in the phenotypic readouts (Figs. 1 and 2).

To further characterize transcriptional dynamics following RIO treatment, we performed pseudo-temporal trajectory analysis using the ImpulseDE package in the TAA model. This analysis revealed that RIO treatment in regression induced expression of *Cyp2c37*, which is associated with hepatocyte metabolic fitness. An increase in the vascular markers *Sema3g* and *Nos3* indicates improvement in sinusoidal structural integrity, coinciding with an upregulation of *Scarf1*, which suggests improved clearance of apoptotic cells. Upregulation of the matrix modulator *Timp2* indicates changes in turnover of ECM in regression.

## Discussion

In this study, we investigated the therapeutic potential of the sGC stimulator RIO to enhance regression of liver fibrosis and PH in two toxin-induced murine liver disease models regressing after cessation of CCl_4_ and TAA induction. By focusing on the regression phase rather than disease progression, this study addresses a clinically relevant, yet understudied therapeutic window.

In both models, key disease parameters, including PH and liver injury markers, largely normalized within 2 weeks of toxin withdrawal. This rapid spontaneous recovery narrowed the therapeutic window and limited our ability to detect additive effects of pharmacological sGC stimulation after 2 weeks of regression. Importantly, after 1 week of regression, portal pressure remained elevated, providing a window for therapeutic intervention. RIO treatment did not significantly accelerate PH resolution at this timepoint; however, numerical reduction in portal pressure was observed. Contrary to PH and liver injury, CPA remained elevated after both 1 and 2 weeks of regression. However, RIO treatment did not reduce fibrosis in both models.

On the transcriptional level, RIO reduced inflammation and improved metabolic function in a model-specific manner. GSEA revealed a reduction of inflammatory pathways, including IL-6–STAT3 and TNF–NFkB signaling in the CCl_4_ model. In the TAA model—in which less differentially expressed genes could be detected—these pathways were not influenced by RIO treatment. Yet ImpulseDE analysis revealed upregulation of Scavenger Receptor Class F Member 1 (*Scarf1*) by RIO, a scavenging receptor which is expressed on endothelial and innate immune cells and which has been implicated to regulate clearance of apoptotic cells and modulation of inflammatory response [30]. Its upregulation may, therefore, reflect enhanced resolution-associated immune functions.

In addition to inflammatory modulation, RIO treatment was associated with enrichment of metabolic pathways, including oxidative phosphorylation, gluconeogenesis, and xenobiotic metabolism. These findings are consistent with improved hepatocellular functional recovery and align with the known role of cGMP signaling in metabolic regulation. Although these transcriptomic changes did not result in statistically significant improvements in fibrosis or portal pressure, they suggest that sGC activation may support restoration of hepatic metabolic competence during regression.

Transcriptionally RIO treatment reduced EMT pathway activity in the CCl_4_ model, which is associated with reduced fibrogenesis. This aligns with an upregulation of Tissue inhibitor of metalloproteinase-2 (*Timp2*) in the TAA model upon RIO treatment. Modulation of EMT pathway and *Timp2* suggests that sGC stimulation may influence matrix remodeling. However, this transcriptional finding did not translate to improvements in collagen proportionate area, which remained unchanged in both models upon RIO treatment in regression.

The efficacy of RIO treatment (i.e., its target engagement) was confirmed by a strong upregulation of hepatic cGMP in the CCl_4_ model in RIO-treated animals. Pathview analysis confirmed an activation of the NO–sGC–cGMP pathway on a transcriptional level, as well as targeted analysis of genes such as *Nos3* in both the CCl_4_ and the TAA models. *Nos3*, encoding endothelial nitric oxide synthase (eNOS), is a central regulator of intrahepatic vascular tone and sinusoidal endothelial function. Increased *Nos3* expression is consistent with activation of the NO–sGC–cGMP axis and may support vascular remodeling during regression. The concomitant upregulation of *Nos3* and Semaphorin-3G (*Sema3g*) indicates improved structural integrity and thus re-capillarization of LSECs upon RIO treatment.

Despite these transcriptional alterations, RIO treatment did not significantly enhance histological fibrosis regression or PH within the observation period. This dissociation between molecular and phenotypic readouts suggests that while sGC stimulation engages relevant vascular, inflammatory, and metabolic pathways, the magnitude or duration of intervention in our models may have been insufficient to translate into measurable structural improvement.

A limitation of this study is the rapid spontaneous regression observed in both murine models. Within 2 weeks, PH and liver injury markers approached levels comparable to healthy controls, thereby restricting the dynamic range for detecting additive therapeutic effects. Although a 1-week regression timepoint was evaluated in the TAA model to capture an earlier intervention window, the effects of RIO remained modest. Prolonged treatment in a model with more sustained residual fibrosis, or in settings of incomplete etiological resolution, could reveal additional therapeutic potential of sGC stimulation.

In conclusion, RIO treatment did not significantly accelerate fibrosis regression or resolution of PH in two murine models of liver disease regression. However, consistent transcriptional modulation of inflammatory, vascular, and metabolic pathways indicates biological activity of sGC stimulation during regression. These findings suggest that pharmacologic enhancement of sGC signaling may influence key regulatory processes during hepatic recovery and may require further investigation in models with prolonged or incomplete regression.

## Supplementary Information

Below is the link to the electronic supplementary material.

Supplementary file1 (DOCX 4740 KB)

## Data Availability

The data that support the findings of this study are available from the corresponding author upon reasonable request.
