# Supplementary material for: Impact of pharmacological sGC stimulation with Riociguat in experimental models of liver disease regression
Source: Hepatol Int. 2026 Apr 15;20(4):1116–26. doi: 10.1007/s12072-026-11086-4 (PMC13518421; doi:10.1007/s12072-026-11086-4)
Supplement: Supplementary file 1 [file 12072_2026_11086_MOESM1_ESM.docx]

**Supplementary figures**

**Supplementary figure 1**

(A) Liver to body weight ratio (CCl_4_ model). (B) Liver to body weight ratio (TAA model). (C) Spleen to body weight ratio (CCl_4_ model). (D) Spleen to body weight ratio (TAA model). (E) Serum bile acids (CCl_4_ model). (F) Serum bile actids (TAA model).

Statistics: One-way ANOVA with Tukey’s multiple comparison test, Welch ANOVA with Dunnett’s T3 multiple comparison test or Kruskal-Wallis test with Dunn’s multiple comparison test were used, after determination of normality by Shapiro-Wilk test.

Significance: * p<0,05; ** p<0.01; *** p<0,001; **** p<0,0001.

Abbreviations: OO (olive oil), CCl_4_ (carbon tetrachloride), TAA (thioacetamide), R2 (two week regression), RIO (riociguat).

**Supplementary figure 2**

(A) Volcano plot of differentially regulated genes between and peak fibrosis and healthy (CCl4 model). (B) Gene set enrichment analysis (GSEA) of differentially regulated genes in fibrosis progression, p.adjust cutoff: 0.05 (CCl4 model). (C) Volcano plot of differentially regulated genes between peak fibrosis and healthy (TAA model). (D) Gene set enrichment analysis (GSEA) of differentially regulated genes in fibrosis progression, p.adjust cutoff: 0.6 (TAA model). (E) Pathview analysis of differentially expressed genes in RIO treatment in regression compared to spontaneous regression for the cGMP-PKG pathway (CCl4 model). (F) Targeted analysis of genes associated with fibrogenesis, angiogenesis and inflammation.
